# Assessment of skin pigmentation-related bias in pulse oximetry readings among adults

**DOI:** 10.1007/s10877-023-01095-1

**Published:** 2023-10-26

**Authors:** Ashish K. Khanna, John Beard, Sakari Lamminmäki, Johanna Närväinen, Nicholas Antaki, Halit O. Yapici

**Affiliations:** 1 Department of Anesthesiology, Section on Critical Care Medicine, Wake Forest School of Medicine, Atrium Health Wake Forest Baptist Medical Center, Winston-Salem, NC USA; 2 Outcomes Research Consortium, Cleveland, OH USA; 3 Perioperative Outcomes and Informatics Collaborative (POIC), Winston-Salem, NC USA; 4 GE HealthCare – Patient Care Solutions, Milwaukee, WI USA; 5 GE HealthCare – Patient Care Solutions, Helsinki, Finland; 6 VTT Technical Research Centre of Finland Ltd, Espoo, Finland; 7 Boston Strategic Partners Inc, Boston, MA USA

**Keywords:** Pulse oximetry, Measurement, Hypoxemia, Racial Bias, Skin pigmentation

## Abstract

**Purpose:**

Recent reports that pulse oximeters may overestimate oxygen saturation in individuals with darker skin pigmentation have prompted concerns from regulatory authorities regarding racial bias. We investigated the performance of TruSignal SpO2 sensors (GE Healthcare, Helsinki, Finland) in adults with varying skin pigmentation.

**Methods:**

A retrospective study was conducted using a set of pooled assessments of SpO2/SaO2 measurements from nine studies to assess bias, accuracy (A_rms_), and precision of TruSignal sensors in healthy adults under induced hypoxia. Subgroup analyses were performed based on oxygen saturation levels (band 1, ≥ 70 and ≤ 80%; band 2, > 80 and ≤ 90%; band 3, > 90 and ≤ 100%).

**Results:**

Of the 10,800 data points from 131 individuals, 8,202 (75.9%) and 2,598 (24.1%) were assigned to the light and dark pigment groups, respectively. Bias was 0.14% overall and less than 1% across oxygenation bands. The difference in bias between dark and light pigment groups was statistically significant at the low oxygenation band with SpO2 ≥ 70 and ≤ 80% (+ 0.58% and + 0.30% respectively; p = 0.0035). Throughout the saturation range, A_rms_ was 1.64% in the light and 1.71% in the dark pigment group, within device specifications and regulatory requirements. Oxygenation was the dominating factor in stepwise ANOVA modeling. The mixed model also showed that bias was strongly affected by the oxygenation range.

**Conclusion:**

TruSignal sensors demonstrated higher bias at lower oxygen saturation, with less than 0.5% difference between pigment groups. These findings raise new questions, such as ways to improve pulse oximetry measurements during challenging clinical conditions, including low perfusion.

## Introduction

Pulse oximeters are non-invasive devices used to approximate a patient’s arterial blood oxygen saturation (SaO2) and pulse rate in multiple settings, including the intensive care unit, operating rooms, and emergency departments, with a recent increased use in homes [1]. These devices feature an optical sensor attached to a patient’s finger, toe, or earlobe [2]. To report the peripheral blood oxygen saturation (SpO2), the level of oxygenated hemoglobin is measured by assessing the transmitted red and infrared light through a volume of tissue. These SpO2 measurements are expected to correlate with the SaO2 values closely; notably, SaO2 is the gold standard for blood oxygenation assessments and is measured from arterial blood samples using multi-wavelength co-oximetry [3, 4]. The importance of pulse oximetry has been well-established and is regarded as a requirement for patient monitoring by the American Society of Anesthesiologists [5].

The performance of a pulse oximeter is typically assessed by evaluating bias, accuracy, and precision. These three interconnected parameters are calculated using data from simultaneously collected SpO2 and SaO2 measurements across oxygenation levels between 70 and 100%. The U.S. Food and Drug Administration (FDA) and the International Organization for Standardization (ISO) 80601-2-61 established accuracy root mean square (A_rms_) thresholds, which are ≤ 3.0% and ≤ 4.0%, respectively [1, 6].

The FDA released a Safety Communication in 2021 expressing concerns that pulse oximeters may be less accurate in certain populations [7], followed by a 2022 report highlighting pulse oximeter measurement errors with increased skin pigmentation from the agency’s Anesthesiology and Respiratory Therapy Devices Panel of the Medical Device Advisory Committee [8]. These were largely in response to the literature reporting higher SpO2 bias and occult hypoxemia in individuals with increased skin pigmentation [4, 9–12]. A recent multicenter retrospective cohort study showed greater bias and higher prevalence of occult hypoxia (i.e., SaO2 < 88% and SpO2 ≥ 92%) in the black patient group compared to white inpatients [13]. In 2022, Shi et al. found that oxygen saturation may be overestimated in individuals with high levels of skin pigmentation and whose ethnicity is reported as Black/African American based on a systematic literature review [4]. The importance of this topic was also highlighted by a 2020 letter authored by Sjoding et al. to the editor of the New England Journal of Medicine, which showed high prevalence of occult hypoxia in black patients, suggesting the need to understand and correct racial bias in pulse oximetry [9].

Therefore, it is critical to evaluate the performance of pulse oximeters for skin pigmentation-related bias or racial bias [14]. GE TruSignal pulse oximetry technology was developed with data acquired from individuals with a wide range of skin pigmentation levels. The technology is intended for continuous SpO2 and pulse rate monitoring of adult, pediatric, and neonatal patients in a hospital environment and during intra-hospital transport. The objective of this study was to investigate the performance of TruSignal SpO2 sensors (GE Healthcare, Helsinki, Finland) with different skin pigmentation levels in healthy adult volunteers during induced hypoxia.

## Methods

### Study design and ethical considerations

A retrospective data analysis was conducted using a set of simultaneous SpO2 and SaO2 measurements from nine separate studies to evaluate bias, accuracy and precision of GE TruSignal SpO2 sensors in adult volunteers under induced hypoxia. The primary aim of the included studies was to evaluate the performance and accuracy of the studied pulse oximeter devices. These studies were designed to assess the device’s accuracy and precision in measuring SpO2 across a range of physiological variables (i.e., oxygenation levels) and subject populations, thus substantiating its suitability for clinical applications. Subjects were healthy, non-smoker, adult volunteers aged 18 to 50 years old. Subjects with any systemic diseases or current health conditions that could increase study risk were excluded from the studies. Study protocols were aligned with the guidelines of Pulse Oximetry standard ISO 80601-2-61 [1], Annex EE.2 “Procedure for invasive laboratory testing on healthy adult volunteers” and FDA Guidance for Pulse Oximeter Pre-Market Notification Submissions [6]. The oxygenation state was manipulated via adjustments to the fraction of inspired oxygen delivered to subjects in order to achieve series of stable SaO2 plateaus at desired saturation levels between 70 and 100% SaO2. Data collection software from the device manufacturer (GE HealthCare, Helsinki, Finland) was used to collect SpO2 data from the test devices with 1 Hz frequency. At each of the SpO2 plateau, arterial blood samples were periodically taken from the radial arterial cannula and analyzed with a calibrated co-oximetry device to perform time-aligned pairwise SaO2 - SpO2 comparisons. An aggregate dataset from the nine above-mentioned studies was utilized and included 10,985 data points from 131 individuals. The final data set for this assessment did not include any subject-identifiable information. Each of the studies was evaluated and approved by an independent institutional review board.

### Outcomes and analyses

The outcome measures were bias (i.e., mean of SpO2 - SaO2), A_rms_ (i.e., accuracy calculated as root mean square of SpO2 - SaO2) and precision (i.e., the standard deviation of SpO2 - SaO2). Data points were assigned a binary pigment group (i.e., light or dark skin pigment) and an oxygenation band based on SaO2 readings (i.e., band 1, ≥ 70 and ≤ 80%; band 2, > 80 and ≤ 90%; band 3, > 90 and ≤ 100%). Skin pigment group was designated using a 5-level classification system: light, medium-light, medium, medium dark and dark [15]. The light pigment group (light, medium light, medium) corresponded to Fitzpatrick I-III and the dark pigment group (medium dark & dark) covered Fitzpatrick IV-V.

To ensure the data quality, exclusion criteria in the study protocols were applied to remove data points related to error conditions in co-oximetry analysis, or data points with total hemoglobin (tHb) < 10 g/dl, methemoglobin (MetHb) > 2% or carboxyhemoglobin (COHb) > 3%, and temporal instability of the SpO2 measurement proximate to the time of obtaining the corresponding blood sample (indicated by a control pulse oximetry). The data collected during motion artefacts or very low perfusion (PI < 0.25%) were also excluded as well as the data points with SaO2 < 70%.

The dataset was inspected using coefficients and p-values for Spearman (Rs) and Pearson (Rp) correlations. Linear and rank correlations, t-tests and SpO2-SaO2 difference plots were utilized for analysis. Furthermore, paired (i.e., SpO2 vs. SaO2 comparisons) and unpaired (in-between group comparisons) two-tailed t-tests were run to investigate differences among oxygenation band subsets of data assuming unequal variance. SpO2-SaO2 difference plots were created using SaO2 as ground truth. The data was initially modeled with ANOVA to assess the interactions among various factors (i.e., pigment group and oxygenation band). Mixed model was used to confirm that the observed effects were valid, and Tukey’s honestly significant difference test was used to analyze the interaction between pigment group and oxygenation band [16]. R (version 3.6; R Foundation for Statistical Computing, Vienna, Austria) was used for mixed models and all other data analyses were performed in MATLAB (The MathWorks® Inc., Natick, Massachusetts, USA version R2020a).

## Results

### Data inspection and descriptive statistics

The correlation and the distribution of the differences in SpO2 - SaO2 is provided in Fig.S1. Overall, 189 data points with SaO2 < 70% were removed, all of which were associated with individuals who self-identified as white without a comparator with different pigmentation, and the final dataset included 10,800 data points from 131 subjects. Of these, 8,202 (75.9%) data points were assigned to the light pigment group and 2,598 (24.1%) to the dark pigment group. Mean age of participants was 25.95 (± 6.57) years and 46.4% (n = 5006) of the data points were from participants identified as female. Detailed characteristics are provided in Tables 1 and 2.

**Table 1:** Demographic information

Characteristics	Light Pigment (n = 8,202, 75.9%)	Dark Pigment (n = 2,598, 24.1%)	Overall^b^ (n = 10,800, 100%)
Age			
Mean, ±SD	26.20 (6.72)	25.16 (6.11)	25.95 (6.57)
Range (lowest, highest)	32 (18, 50)	23 (18, 41)	32 (18, 50)
Sex, n (%)			
Female	4412 (40.9)	594 (5.5)	5006 (46.4)
Male	3790 (35.1)	2004 (18.6)	5794 (53.6)
Race, n (%)			
White	5500 (50.9)	135 (1.3)	5635 (52.2)
Asian	320 (3.0)	326 (3.0)	646 (6.0)
Black/African American	0 (0.0)	1540 (14.3)	1540 (14.3)
Other^a^	2382 (22.1)	597 (5.5)	2979 (27.6)

^a^Other includes American Indian, Alaskan Native, Native Hawaiian, Other Pacific Islander, and Not Asked

^b^The 10,800 data points were collected from 131 subjects

n, number of samples; SD, standard deviation

**Table 2:** Skin pigmentation level distribution

Skin Pigmentation Level	Number of samples, n (%)
1 (Light)	4399 (40.7)
2 (Medium-Light)	1317 (12.2)
3 (Medium)	2486 (23.0)
4 (Medium-Dark)	605 (5.6)
5 (Dark)	1993 (18.5)
Total	10,800 (100.0)

### Analysis of bias

**Fig. 1:**
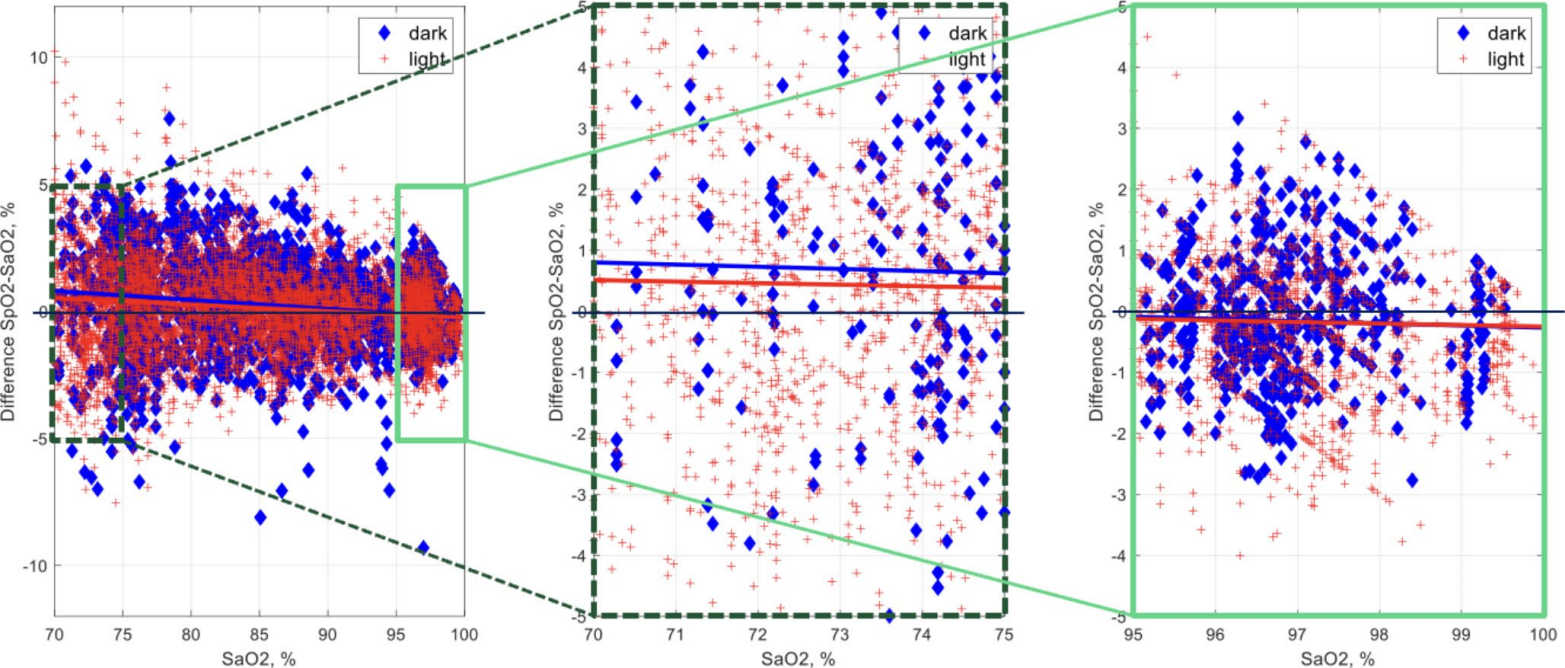
Linear correlation fitted separately for dark and light pigment groups (left) and zoom-ups of low (70–75%) and high (95–100%) oxygenation ends (middle and right)

Overall, the SpO2 and SaO2 oxygenation readings were largely in agreement and the largest observed disagreements were around 10% (i.e., maximum of 10.2%); 99.1% of the differences between observed SaO2 and SpO2 were within -/+ 5% and 92.3% of the differences were within -/+3%. The difference for all data points in dark and light groups are plotted in Fig. 1.

**Fig. 2:**
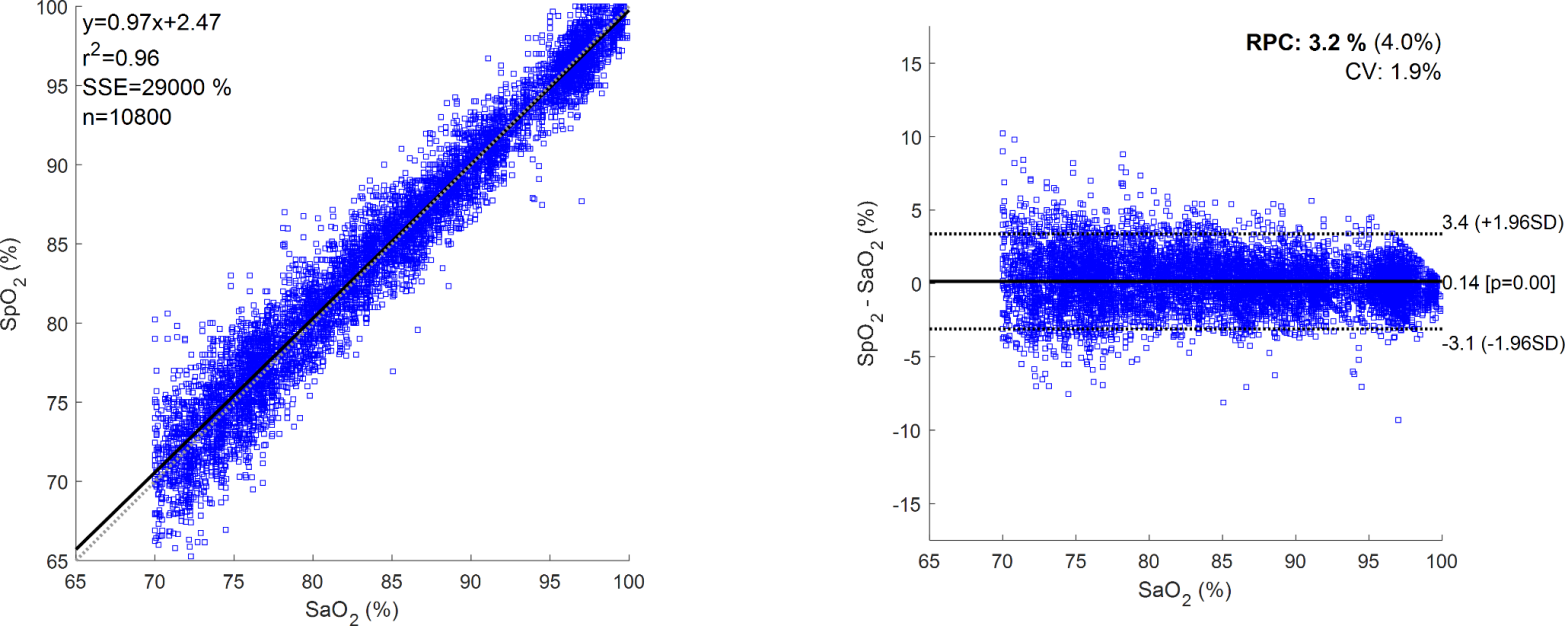
Correlation (left) and SpO2-SaO2 difference (right) plots for all data

Correlation and SpO2-SaO2 difference plots demonstrated minor differences in bias between light and dark pigmentation groups, which were small compared to variations within either group alone (Fig. 2, S2 and S3). The overall mean bias was equal to 0.14%, indicating a slight overall overestimation of oxygenation. The oxygenation-band dependent bias (i.e., deviation from the line of unity, indicated with a dotted line on the correlation plot) was minor compared to the variation.

### Subgroup analysis based on oxygenation bands

Bias, accuracy, and precision by oxygenation band were reported for each pigment group in Table 3. When looking at individual oxygenation bands, the only statistically significant difference in bias between the dark and light pigment groups was found at band 1 (i.e., ≥ 70 and ≤ 80%), indicating a slightly larger bias for the dark pigment group (p = 0.0035; Table 3 and Fig.S4). At high oxygenation (i.e., > 90 and ≤ 100%), bias was slightly negative for each pigment group, suggesting underestimation of oxygenation. Mean bias was less than 1% across all oxygenation bands with a whole range p-value of 0.0370 driven by the difference observed in band 1. In both pigment groups, A_rms_ was below 2% in the entire oxygenation range, and well below 3% for each separate oxygenation band.

**Table 3:** Performance of SpO2 measurements for Dark, Light and All groups

Oxygenation	Bias, %				Precision, %				Accuracy (A_rms_), %			
	Dark	Light	All	P-value		Dark	Light	All		Dark	Light	All
**Whole range**	0.20	0.12	0.14	0.0370		1.70	1.63	1.65		1.71	1.64	1.66
**> 90 and ≤ 100%**	-0.14	-0.17	-0.16	0.4765		1.18	1.18	1.18		1.19	1.19	1.19
**> 80 and ≤ 90%**	0.30	0.24	0.25	0.2699		1.67	1.48	1.53		1.69	1.50	1.55
**≥ 70 and ≤ 80%**	0.58	0.30	0.35	0.0035		2.26	2.09	2.13		2.33	2.11	2.16

### Multivariate and mixed models

Stepwise n-way ANOVA modeling was utilized to analyze the data as a whole and estimate the contribution of each factor (i.e., pigment group and oxygenation band) and their interaction (Table S1). In the model, oxygenation was the dominating factor, pigmentation was significant as well, and their interaction contributed to the model. This suggests that when all data points are treated as independent samples, the overall bias depends heavily on the oxygenation range, and there is a weak pigment bias depending on the oxygenation range studied.

ANOVA neglects the fact that the data contains several samples from each participant. To address this, the data was also processed using a mixed model shown in Table S2. Subject was initially taken as a random factor, after which different factors were separately added to the model. Unlike in ANOVA modeling, the mixed model did not improve significantly after adding the skin pigment group factor, but it was enhanced when including the oxygenation factor. Combining skin pigment and oxygenation factors did not refine the model compared to oxygenation alone. On the other hand, combining skin, oxygenation, and their interaction slightly improved the model over oxygenation alone (p = 0.0252). The mixed model confirmed that even when subject is included as a random factor, bias is strongly affected by the oxygenation range, and skin pigment has a weak effect with significance that depends on the oxygenation range.

## Discussion

This large retrospective data analysis assessed data from nine different studies and evaluated over 10,000 data points to investigate skin pigmentation-related bias in pulse oximetry readings. Overall, we found low bias, high accuracy, and high precision in the study device. Device performance was consistent across varying levels of oxygen saturation and skin pigmentation. The single statistically significant difference in bias of 0.28% was between the dark and light pigment groups at band 1 (i.e., ≥ 70 and ≤ 80%) and no significant differences were found in precision or A_rms_. Worth noting, the bias for the whole range had a p-value lower than 0.05, but was not significant following a Bonferroni correction, after which the significance threshold was lower (needed p-value of 0.05/4 = 0.0125) because of multiple comparisons.

Our research is timely and important, not only because of the recent and extensive body of literature suggesting racial bias in pulse oximeters, but also considering the interest expressed by multiple regulatory authorities. Previous literature evaluated pulse oximeter readings and reported higher bias in subjects with dark pigmentation [4, 11, 12], which prompted regulatory scrutiny and an urgent effort to clarify the association between skin pigmentation and oximeter accuracy [7, 13, 17]. Given the widespread use of SpO2 in clinical decision making, any systemic differences between SpO2 and SaO2 could have major health implications [18] and could disproportionately impact individuals with darker skin pigmentation.

Overall, the differences in bias between pigment groups found in this study are less than previous literature [12]. In our analyses, biases were of lower magnitude than the corresponding variances accuracy and precision values, which indicates that the systematic under- or overestimation of the oxygenation state was less than the overall variation in the measurement. Moreover, A_rms_ values in all oxygenation bands investigated were within regulatory standards, including below the 3% and 4% limits of the FDA guideline [6] and ISO standard requirements [1] respectively. The accuracy trended lower in the dark pigment group but the difference between dark and light pigment groups was not statistically significant. Considering the above-mentioned concerns regarding the skin pigmentation-related bias, lower accuracy uniformly across pigment groups is not as contributory as the difference in precision or bias between pigment groups that could disproportionately influence clinical care decisions.

Our results were largely similar when subgroups of oxygenation bands were analyzed as recommended by the FDA [6]. In the lower oxygenation band (i.e., ≥ 70 and ≤ 80%), performance declined in both pigment groups and bias was significantly higher in the dark pigment population. However, the bias and its difference between the two pigment groups were relatively small compared to A_rms_. Increased bias is concerning, especially in lower oxygenation levels, since these measurements may impact clinical decisions and therapies in a relatively sicker population. Nevertheless, these findings were consistent with the widespread literature reporting a deterioration of pulse oximeter performance in low oxygenation levels [11, 19, 20]. In fact, most clinicians recognize that SpO2 accuracy worsens in serious hypoxia, and do not solely rely on SpO2 readings for clinical decision-making.

Another interesting finding was the limited occurrence of occult hypoxemia in our dataset (i.e., SaO2 < 88% and SpO2 between 92% and 96%). In both the dark and light pigment groups; there was only one observation in the dark pigment group fulfilling these criteria with SpO2 of 92.0% and SaO2 of 87.4%. This contrasts with the results of a large study published in 2020 that evaluated the performance of pulse oximetry in patients self-identified as Black and White in the critical care setting. Although different from our assessment that included healthy individuals, the study demonstrated that the frequency of clinically significant occult hypoxemia was around three times more in Black (11.4%) than in White (3.6%) patients [9]. Undetected hypoxemia may exacerbate clinical outcomes as recent studies showed that the critically ill with occult hypoxemia have higher mortality and greater incidence of organ failure [13, 21, 22]. It was also associated with delayed or non-administration of therapies in patients with COVID-19 [23]. Therefore, higher incidence of occult hypoxemia in patients of racial and ethnic minority groups could possibly lead to insufficient treatment and contribute to known disparities in outcomes, including those seen during the COVID-19 pandemic [22]; it is important to limit its occurrence as was the case in our findings, and ensure there is no clinically meaningful difference across skin pigmentation groups.

Despite an extensive body of literature revealing widespread skin pigmentation-related bias in pulse oximeters, we reported low bias with the study device in adults, in both light and dark pigment groups. Another controlled hypoxia study published by Barker et al. in 2022 evaluated distinct pulse oximetry sensors and found no clinically significant differences in bias between healthy Black and White subjects [24]. However, both our study and Barker et al. (2022) evaluated device performance in healthy adults, which may not be comparable to previous studies on inpatients. In fact, one major factor that may affect the comparability is the presence of low peripheral perfusion in hospitalized patients [24]. Prospective real-world studies are needed to obtain the full picture of TruSignal SpO2 performance. The Anesthesiology and Respiratory Therapy Devices Panel also highlighted the need for additional studies of patients in health care settings to better reflect real-world performance of pulse oximeters [12].

The difference in performance of pulse oximeters seen across various studies may stem from nuances in device design. Technology may play a role in overcoming skin pigmentation-related bias and thus devices should be designed, calibrated, and verified taking these concerns into consideration. Even though all commercial pulse oximeters rely on a similar technology based on dual-wavelength spectrophotometry, implementation details may affect skin pigmentation-related bias. In fact, these differences may be related to SpO2 sensor’s optical/mechanical design, signal acquisition electronics, control software and signal processing. Dark pigmentation increases signal attenuation particularly in red wavelength and therefore requires additional design considerations (e.g., spectral characteristics of optical components, LED driver and receiver dynamic range, system signal to noise ratio [SNR] and system linearity). While the study device also functions based on dual-wavelength spectrophotometry with red/infrared wavelengths, it features several characteristics, which may partially explain our findings. To maintain a high SNR with different tissue pigmentations, the device’s signal levels related to the two LEDs can be adjusted independently. Furthermore, special considerations were taken in the sensor and receiver design to maintain system linearity across the wide dynamic range required with dark pigmented skin. In addition, the effect of ambient light is minimized with dedicated filtering techniques. Finally, applied algorithms may play a role in reducing bias as they analyze the signal and filter out effect of motion artefact and contribution of low perfusion. An empirical calibration equation is then used to convert the amplitude ratio of red and infrared signals to SpO2 value.

Future device development should take our findings, combined with the previous literature, into consideration since medical devices that may disproportionately impact patient groups are an important regulatory and clinical issue. Comprehensive studies are needed to better understand the specific device features that might affect bias across different pigmentation levels. Pulse oximetry technologies should be verified/validated under various real-world clinical situations, including anemia, motion, low perfusion, and varied skin pigmentation.

One of the limitations of our analysis is that it evaluated data from nine separate studies presenting some variability in methodology such as differences in protocols and patient monitor used. Pooling data might also have led to limitations associated with retrospective research. Inherent limitations of the available variables (e.g., sex, age, ethnicity) provided across studies also limit our assessment regarding other potential covariates, such as the effect of finger size or peripheral perfusion. However, we utilized appropriate statistical methods to minimize the impact of heterogeneity and to conduct robust analyses. Moreover, the controlled desaturation studies with healthy volunteers have their well-known limitations as induced hypoxia might not replicate a real disease situation, and study findings may not be generalized to hospitalized patients. Indeed, healthy subjects are not prone to several clinical challenges to SpO2 readings, including changes in breathing, anemia, abnormal extremity perfusion, which are more frequent in critically ill patients [24]; nevertheless, the elimination of these known confounders allows for a more targeted assessment of skin pigmentation. Known pulse oximeter confounders including low tHb as well as elevated COHb and MetHb were excluded in our analysis. Furthermore, it is important to emphasize that from an ethical standpoint, desaturation studies can only be conducted with healthy participants in a controlled laboratory setting [24]. Finally, our study included an objective measurement of skin pigmentation (i.e., Fitzpatrick scale) to fully evaluate the effect of melanin variation in pulse oximetry readings and did not rely on self-identified ethnicity/race that may not accurately characterize skin tone.

## Conclusion

Our study assessed bias, accuracy, and precision in TruSignal pulse oximeters using data from nine studies. Our findings demonstrate that the performance of these devices meet medical device standards and that limited differences exist between pigment groups, providing timely evidence related to the ongoing concern from regulatory authorities regarding racial bias in pulse oximeter devices. Further research is needed to evaluate the real-world performance of these devices and to answer key questions regarding specific design elements that may lead to performance improvements.
